# Impact of molecular tumor board on clinical outcomes in patients with refractory solid tumors: a real-world study

**DOI:** 10.1093/oncolo/oyaf196

**Published:** 2025-07-01

**Authors:** Yan Ling, Xiao-Dong Jiao, Bao-Dong Qin, Jiang-Shui Ding, Zhan Wang, Ke Liu, Wen-Xing Qin, Ying Wu, Li- Sun, Dong-Min Shi, Shi-Qi Chen, Xue Zhong, Xiao-Peng Duan, Bing Li, Yuan-Sheng Zang

**Affiliations:** Department of Medical Oncology, Changzheng Hospital, Naval Medical University, Shanghai, 200072, China; Department of Medical Oncology, Changzheng Hospital, Naval Medical University, Shanghai, 200072, China; Department of Medical Oncology, Changzheng Hospital, Naval Medical University, Shanghai, 200072, China; Department of Orthopaedics, Qingyang County People’s Hospital, Anhui, 242800, China; Department of Medical Oncology, Changzheng Hospital, Naval Medical University, Shanghai, 200072, China; Department of Medical Oncology, Changzheng Hospital, Naval Medical University, Shanghai, 200072, China; Department of Medical Oncology, Changzheng Hospital, Naval Medical University, Shanghai, 200072, China; Department of Medical Oncology, Changzheng Hospital, Naval Medical University, Shanghai, 200072, China; Department of Medical Oncology, Changzheng Hospital, Naval Medical University, Shanghai, 200072, China; Department of Medical Oncology, Changzheng Hospital, Naval Medical University, Shanghai, 200072, China; Department of Medical Oncology, Changzheng Hospital, Naval Medical University, Shanghai, 200072, China; Department of Medical Oncology, Changzheng Hospital, Naval Medical University, Shanghai, 200072, China; Department of Medical Oncology, Changzheng Hospital, Naval Medical University, Shanghai, 200072, China; Burning Rock, Department of Medical, Biotech, Guangzhou, 510300, China; Department of Medical Oncology, Changzheng Hospital, Naval Medical University, Shanghai, 200072, China

**Keywords:** refractory solid cancer, molecular tumor board, next-generation sequencing, prognosis

## Abstract

**Introduction:**

Providing precise oncologic treatment for patients with refractory solid tumors is still an unmet need in clinical practice. This study aimed to assess whether treatments recommended by a molecular tumor board (MTB) can improve clinical outcomes in patients with refractory solid tumors.

**Methods:**

We screened all patients with refractory solid tumors during the period from 2017 to 2022 at the authors’ center. The patients with actionable molecular alterations (mainly including druggable tier 2 genetic variants identified using next-generation sequencing [NGS]) were presented to MTB. We compared the overall survival (OS) and progression-free survival (PFS) between the patients treated with a matched therapy recommended by MTB and those who did not receive the MTB-recommended therapy. Patients with no actionable molecular alterations served as an additional control.

**Results:**

A total of 338 patients with refractory solid tumors were screened. Among the 305 patients for whom NGS testing was conducted, 217 patients available for survival outcomes were included in the final analysis. A total of 129 patients had at least one actionable molecular alteration and were presented to MTB; 82 received the MTB-recommended therapy, while the remaining 47 did not. Those who received the recommended therapy had significantly longer median OS (17.7 vs. 4.4 months; HR 0.31, 95% CI: 0.14-0.66; *P* < .001) and median PFS (7.0 vs. 2.3 months, HR 0.32, 95% CI: 0.16-0.65; *P* < .001).

**Conclusions:**

MTB improves oncologic prognosis in patients with refractory solid tumors, and matching MTB-recommended therapy is an independent factor for OS and PFS.

Implications for Practice The molecular tumor board (MTB) has been widely recognized for its importance in precision oncologic therapy. Due to the lack of standard treatment, patients with refractory cancer are in a dilemma with poor survival outcomes. In this real-world cohort study, we sought to determine whether patients with refractory solid cancer benefit from the MTB-recommended optimal treatment strategy. We found that MTB improved oncologic prognosis in patients with refractory solid tumors. Furthermore, matching MTB-recommended therapy is an independent factor for OS and PFS, both with and without immunotherapy-based regimens.

## Introduction

Advances in modern oncology have significantly enhanced disease control and consequently improved the quality of life of cancer patients. Due to the increasing availability of highly effective regimens with low toxicity in the front-line setting, additional lines of treatment are often available for patients with refractory solid tumors upon disease progression.^1^ Establishing the appropriate treatment is critical for optimizing prognosis in such patients, especially when they have additional risk factors for poor outcomes, such as old age and poor organ function. Patients without appropriate treatment regimens, in contrast, could only be treated by supportive care or with alternative medicines. Thus, there is an urgent need to provide precise treatment for patients with refractory cancer.^2-5^

High-throughput sequencing technologies, such as next- generation sequencing (NGS), have radically transformed the treatment of cancer.^6^^,^^7^ Broadening the utility of genome-driven personalized oncology treatment has been emphasized in refractory cancer. However, although remarkable changes in the diagnosis and clinical management of multiple malignancies have been achieved, the benefit from precision therapy is limited in clinical practice due to insufficient understanding of actionable molecular alterations and relevant pathway aberrations, leaving the majority of patients without clinical improvement. Despite the definition of priority genomic alterations, systemic translation of the information obtained from NGS into decision-making in clinical practice is often beyond the understanding of most single physicians. Multiple lines of previous treatment represent another layer of difficulty due to the complex genome background and acquired resistance. In such patients, a one-mutation-one-drug approach may not always be effective.^8^^,^^9^

To bridge the gap between the diverse spectrum of expertise and oncologists for the application of the genomic information to clinical practice, a few cancer centers have established the molecular tumor board (MTB), which generally consists of oncologists, pathologists, bioinformaticians, molecular biologists, and oncology pharmacists.^10^ The MTB model is a powerful tool to comprehensively outline patient characteristics and discuss all potential therapeutic strategies, based on clinical and treatment history, pathology, imaging, laboratory results, and molecular testing. Recently, there has been increasing evidence that MTBs could successfully improve patient prognosis.^11-16^ However, given the lack of reproducibility data, it is still unclear whether patients with refractory cancer could benefit from MTB treatment decisions.

Therefore, we conducted this study to describe the real-world implementation of appropriate combinations of tailored agents based on MTB (eg, targeted, hormonal, chemotherapy, or immunotherapy agents) for patients with refractory solid tumors in Shanghai Changzheng Hospital. We compared the survival between the patients who underwent the MTB-recommended treatments and those who did not, to further determine the impact of MTB on clinical outcomes in patients with refractory solid tumors.

## Materials and methods

### Patients

In the current study, we screened all patients with refractory solid tumors who had been admitted to our center during the period between February 2017 and November 2022. The patients for whom NGS data and survival data were available were included in the final analysis. The last follow-up date was October 31, 2022.

This study was approved by the Ethics Committee of Changzheng Hospital (Shanghai, China). The requirement for informed consent was waived by the Ethics Committee because patients or their legal guardians, at the time of treatment, provided written consent for their medical records to be analyzed and published for research purposes on the condition of anonymity.

### NGS and other molecular analyses

NGS was performed using circulating tumor DNA (ctDNA) extracted from fresh tumor biopsy samples or peripheral blood. If these samples were unavailable, NGS was performed using the most recent tissue sample, such as paraffin- embedded tumor sections, pleural effusion, or ascites. The sequencing was carried out on a platform certified by the Clinical Laboratory Improvement Amendments. Briefly, target capture was performed using a commercial panel consisting of 520 genes (OncoScreen Plus), spanning 1.86 megabases of the human genome. The quality and the size of the fragments were assessed by a high-sensitivity DNA kit using Bioanalyzer 2100 (Agilent Technologies, CA, USA). Indexed samples were sequenced on Nextseq 500 (Illumina, Inc., CA, USA) with paired-end reads and average sequencing depth of 1000× for tissue samples and 10 000 × for liquid biopsy samples. Tumor mutation burden (TMB) was calculated as previously described.^17^ Somatic/germline variants were categorized into 4 tiers based on the consensus recommendations of the Association for Molecular Pathology^6^ and the American Society of Clinical Oncology.^18^ In addition, PD-L1 expression was quantified by tumor proportional score or combined proportional score. HER2 expression was detected using immunohistochemistry (IHC) and further verified by immunofluorescence (IF).

### Recommendations by the MTB

**Figure 1.**
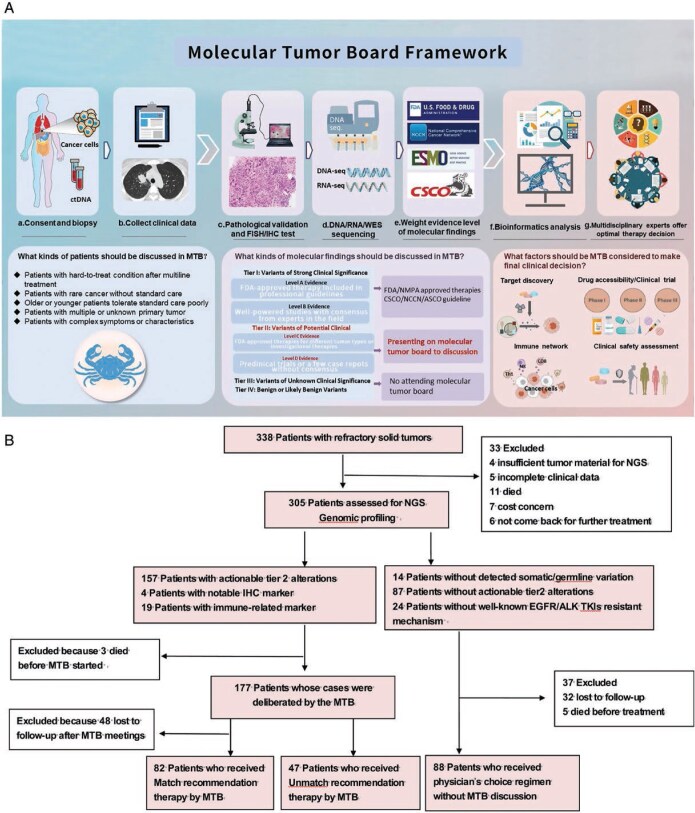
Framework of the MTB and study design. (A). Flowchart of patient enrollment and analysis. (B). Framework of analysis and decision-making by the MTB in the present study. ASCO, American Society of Clinical Oncology; ctDNA, circulating tumor DNA; FDA, US Food and Drug Administration; FISH, fluorescence in situ hybridization; IHC, immunohistochemistry; MTB, molecular tumor board; NCCN, National Comprehensive Cancer Network; TKI, tyrosine kinase inhibitor.

The MTB meetings at our center were convened twice every month and were attended by oncologists, pathologists, bioinformatics specialists, clinical pharmacists, and cancer biologists. During each session (typically 2-2.5 hours), 1-3 cases were discussed in detail in terms of clinical demographics, pathology, imaging, sequencing, and other molecular tests (**Figure 1A**). Cases were referred to the MTB if (a) NGS indicated at least one actionable tier 2 genetic alteration; (b) Eastern Cooperative Oncology Group status was 0-3; (c) the patients had adequate organ function; and (d) life expectancy exceeded 3 months. Patients with high PD-L1 expression (>50%), TMB-H (TMB ≥ 20 mutations/Mb), or MSI-H/dMMR (MHL1, MSH2, MSH6, and/or PMS2 alterations)^19^ were also considered actionable and submitted to the MTB.

Actionable tier 2 alterations included the following: *EGFR* mutation/amplification targeted by TKIs, *ALK* fusion, *BRAF V600E* mutation,^20^  *BRCA1/2* mutation, *CDK4/6* amplification, *CDKN2A/2B* deletion mutation, *FGFR1/2/3* fusion, *MET* amplification/mutation, *ERBB2* amplification/mutation, *PIK3CA* mutation, *IDH1* mutation, *RET* fusion,^21^  *ROS1* fusion, and *NTRK* fusion/rearrangement.^3^^,^^22^ Our MTB considered *TP53* structural mutations as actionable tier 2 alterations. Other *TP53* mutations were considered non-actionable.^23^

### Final treatment decisions

The attending physicians and patients or their legal guardians made the final decision about treatments based on consideration of the MTB recommendations as well as a comprehensive assessment of the patient’s condition. For the purposes of the present analysis, the patients were classified into the following 3 groups: those who were referred to the MTB and received MTB-recommended therapy (the matched group), those who were referred to the MTB but did not receive MTB-recommended therapy (the unmatched group), and those who were not referred to the MTB (the no-marker group).

### Outcomes and statistical analysis

Data were reported as mean ± standard deviation if normally distributed or as median (interquartile range, IQR) if skewed. Categorical data were compared using the chi-square test or Fisher’s exact test as appropriate. Overall survival (OS) was defined as the time from the start of therapy after MTB deliberation until the last follow-up or all-cause death. Progression-free survival (PFS) was defined as the time from the start of therapy after MTB deliberation until the date of disease progression. PFS1 was defined as PFS for the most recent prior therapy. PFS2 was defined as PFS for the most recent therapy after MTB. The ratio PFS2 / PFS1 ≥ 1.3 was taken to indicate treatment benefit.^24^ Treatment response and progression were assessed in accordance with the RECIST 1.1 criteria.^25^ Kaplan–Meier survival curves were compared between the patient groups using the log-rank test. Where appropriate, results were reported in terms of hazard ratios and 95% confidence intervals (CIs). Two-sided *P* < .05 was considered statistically significant. All statistical analyses were conducted using SPSS 16.0 (IBM, Chicago, IL, USA).

## Results

### Patients

A total of 338 patients with refractory solid tumors were screened. Among them, 33 were excluded due to the lack of NGS data, and another 88 were excluded due to the loss to follow-up (**Figure 1B**). The remaining 217 patients (90 women) were included in the final analysis. The median age was 60 years (range, 18-90 years). All but 14 of these patients received at least one line of therapy, whereas 144 (66%) received at least 3 lines of therapy. Among the 14 patients who were treatment-naïve, 3 had an unknown primary tumor, 3 had multiple primary tumors, 3 were older than 75 years, and 1 had a rare cancer without standard-of-care treatment (**Table 1**).

**Table 1.** Baseline clinical demographic characteristics of patients with refractory solid tumors.

**Baseline Characteristics^a^**	**Refractory tumor analysis cohort**	**Patients attending MTB discussion**		**No marker group**	***P*-value**
		**Matched therapy group**	**Unmatched therapy group**		
Patients No.(%)	217(100)	82(37.8)	47(21.7)	88(40.6)	
Age(years,range)	60(18-90)	59(18-89)	59(34-88)	60(20-90)	.38
≥60	116(53.5)	45(54.9)	21(44.7)	50(56.8)	
<60	101(46.5)	37(45.1)	26(55.3)	38(43.2)	
Gender	127(58.5)	51(62.2)	29(61.7)	47(53.4)	.45
Male	90(41.5)	31(37.8)	18(38.3)	41(46.6)	
Female					
Previous lines of	73(33.6)	29(35.4)	19(40.4)	25(28.4)	.34
therapy	144(66.4)	53(64.6)	28(59.6)	63(71.6)	
<3					
≥3					
TMB(Mut/Mb)	172(79.3)	59(72)	33(70.2)	80(90.9)	.01
0-10/Unknown	28(12.9)	12(14.6)	8(17.0)	8(9.1)	
10-20	19(8.8)	11(13.4)	6(12.8)	0(0)	
≥20					
Primary tumor location	74(34.1)	27(32.9)	13(27.7)	34(38.6)	.12
Lung	45(20.7)	17(20.7)	13(27.7)	15(17.0)	
Colorectal	31(14.3)	15 (18.3)	5(10.6)	12(13.6)	
Stomach	8 (3.7)	2(2.4)	4(8.5)	2(2.3)	
Bile duct and gallbladder	7(3.2)	1 (1.2)	0(0.0)	6(6.8)	
	7(3.2)	1(1.2)	1(2.1)	5(5.7)	
Pancreas	9(4.1)	2(2.4)	1(2.1)	6(6.8)	
Esophagus cancer	6(2.8)	4(4.9)	1(2.1)	1(1.1)	
Gynecological oncology	4(1.8)	2(2.4)	1(2.1)	1(1.1)	
Unknown primary	19(8.8)	11(13.4)	3(6.4)	5(5.7)	
Multiple primary					
Others^b^					

a. Values are *n* (%) or median (range); b. Others tumor location includes sarcoma (*n* = 6),Breast(*n* = 4), neuroendocrine neoplasm (*n* = 4), nasopharynx (*n* = 2), paget’s disease (*n* = 2), parotid gland (*n* = 1), Thyroid (*n* = 2), Small intestinal (*n* = 1), perioneal (*n* = 1), Urothelial(*n* = 3).

Among the 217 patients in the final analysis, 129 patients had at least one genetic finding (mainly tier 2 mutations) and were referred to the MTB (**Figure 1A**). Among them, 82 patients received the MTB-recommended therapy, whereas 47 did not. The most frequent reasons for not receiving the recommended therapy were the preference of the patient or attending physician (35 patients: 74.5%), deterioration of the patient’s condition leading to medical care at a local hospital (6 patients: 12.8%), and inaccessibility of the therapy because of cost or availability issue (6 patients: 12.8%).

The remaining 88 patients had no actionable molecular alterations, and their treatment was decided jointly by the attending physician and patient or legal guardian, without involving the MTB. The 3 groups did not differ significantly in age, sex, cancer type, TMB, or lines of previous therapies (**Table 1**, **Supplementary Table 1**).

### Molecular characteristics of tumors

**Figure 2.**
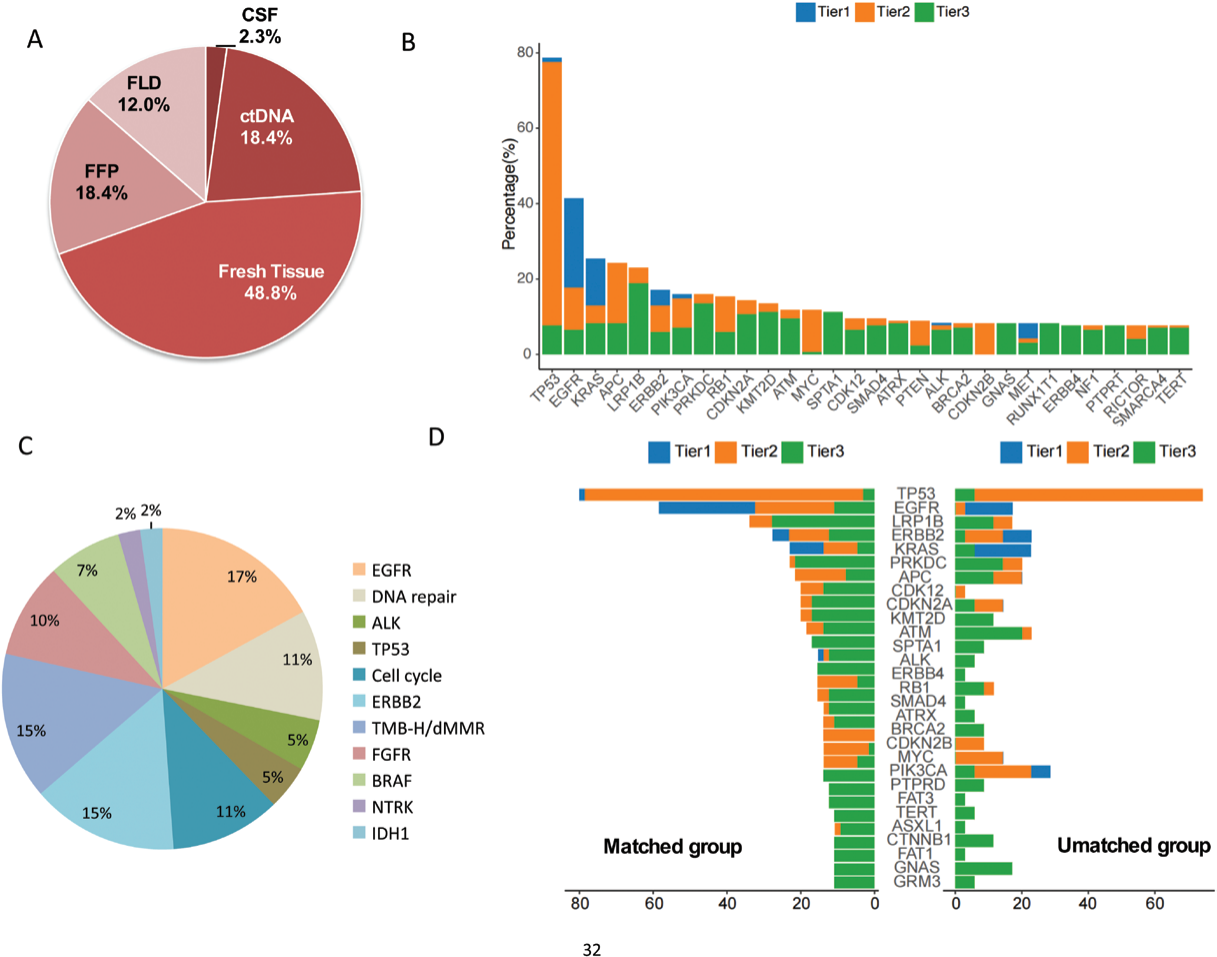
Genomic alterations in refractory solid tumors. (A) Sources of sample for NGS. (B) Distribution of the 30 most frequent genomic alterations in tissue NGS. Alterations were classified by tier in accordance with the Association for Molecular Pathology guidelines. (C) Summary of actionable genes and signaling pathways most frequently discussed in the MTB to inform treatment strategies. ‘EGFR’ refers to EGFR mutation and amplification; ‘ALK’ refers to *ALK* fusion; ‘ERBB’ refers to *ERBB2* amplification/mutations or HER2 positivity as assessed by IHC; ‘TMB-H/dMMR’ refers to tumor mutation burden high/mismatch repair deficiency; ‘FGFR’ refers to *FGFR1/2/3* fusion; ‘Cell cycle’ refers to *CDK4/6* amplification or *CDKN2A/2B* deletion mutation; ‘DNA repair’ refers to *BRCA1/2* mutation or high HRD score; ‘BRAF’ refers to *BRAF V600E* mutation; ‘IDH1’ refers to *IDH1* mutation; ‘NTRK’ refers to *NTRK* fusion/rearrangement. (D) Comparison of the most frequent genomic alterations in patients who were referred to the MTB between the matched group and the unmatched group, sorted by tier grade. CSF, cerebrospinal fluid; FLD, tissue fluid; FFP, formalin-fixed paraffin.

NGS was conducted most often using ctDNA from fresh tumor biopsy specimens, followed by peripheral blood samples, paraffin-embedded tumor sections, and cerebrospinal fluid or pleural effusion (**Figure 2A**). NGS data were available for both tumor tissue and ctDNA in 66% (144/217) of the patients. The average number of alterations per patient was 12.6 mutations (cfDNA: 12.12 mutations; tissue: 12.69 mutations). Of the 98 patients for whom PD-L1 expression data were available, 59 (60%) were positive for PD-L1. TMB-high (≥20 mutations/Mb) and MSI-high were identified in 8.8% (19/217) and 1.8% (4/217) of the patients, respectively. The most common mutations included *TP53* (71.8%) and *KRAS* (21.8%; **Figure 2B**). Oncogenic mutations included *EGFR* (21.2%), followed by *ERBB2* (12.4%) and *PIK3CA* (13.5%; **Figure 2B**). The ctDNA mutation profiles were similar to tissue NGS (**Supplementary Figure 1**).

Among the 129 patients whose cases were referred to the MTB, the 5 most frequent aberrant signaling pathways were mutations in *EGFR* (23, 17%), mutation or amplification of *ERBB2* (20, 15%), TMB-high or dMMR (20, 15%), defects in DNA damage repair (15, 11%), and mutation or deletion of genes involved in the cell cycle (15, 11%; **Figure 2C**). The mutation profiles of the matched group vs. the unmatched group are shown in **Figure 2D**. The genetic aberrations were classified as tier 1, 2, and 3/4, including 69 (3.6%), 371 (19.4%), and 1474 (77.0%) cases, respectively. The percentage of tier 2 gene alterations was 19.3% in the matched group and 19.6% in the unmatched group. Tier 2 gene alterations noted by the MTB as the basis for a potential clinically actionable target are described in the Materials and Methods. The therapies recommended by the MTB and the response to those treatments are listed in the **Supplementary Material**. **Table 1.** Baseline clinical demographic characteristics of patients with refractory solid tumors.

### Survival outcomes

**Figure 3.**
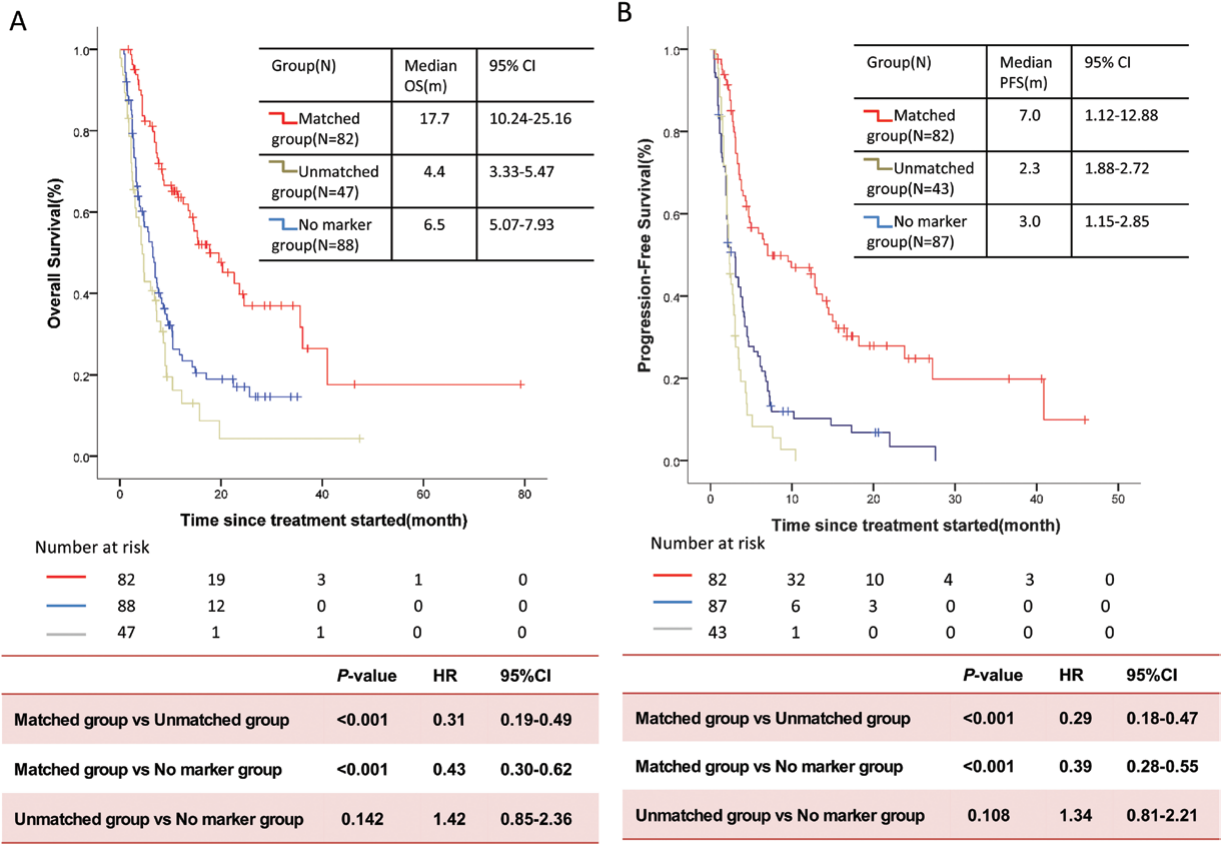
Overall survival and progression-free survival. Kaplan–Meier curves of (A) overall survival or (B) progression-free survival among patients in the matched group, the unmatched group, and the no-marker group.

The median follow-up was 22.3 months (IQR, 12.1-31.9 months) for the entire cohort. The median OS was 17.7 months (95% CI: 10.24-25.16) in the matched group and 4.4 months (95% CI: 3.33-5.47) in the unmatched group (**Figure 3A**). The median OS in the no-marker group was 6.5 months (95% CI: 5.07-7.93). The HR of all-cause death in the matched group was 0.31 (95% CI: 0.19-0.49; *P* < .001) in comparison with those not receiving MTB-recommended treatment. The risk did not differ between the patients in the unmatched group and those in the no-marker group (HR, 1.42; 95% CI: 0.85-2.36; *P* = .142).

Multivariate Cox regression showed that among the patients whose cases were deliberated by the MTB, receiving matched therapy was independently associated with favorable OS relative to the patients who received the unmatched therapy (adjusted HR, 0.37; 95% CI, 0.25-0.55; *P <* .001; **Table 2**). Receiving the matched therapy was also independently associated with favorable OS relative to the patients who were not referred to the MTB (adjusted HR, 0.39; 95% CI: 0.26-0.58; *P* < .001; **Table 2**).

**Table 2.** Survival of patients with different characteristics (entire cohort).

**Patient characteristics**	**OS**						**PFS**					
	***N***	**Median(month) (95%CI)**	**Univariate Cox model HR (95%CI)**	***P*- value**	**Multivariate Cox model HR (95%CI)**	***P*- value**	***N***	**Median(month) (95%CI)**	**Univariate Cox model HR (95%CI)**	***P*- value**	**Multivariate Cox model HR (95%CI)**	***P*- value**
Age(years)	116	9.2 (7.36-11.04)	0.81(0.58-1.11)	.187	0.91(0.32-1.62)	.617	101	4.1(2.86-5.34)	0.83(0.62-1.12)	.227	0.97(0.69-1.35)	.833
≥60	101			——		——	111	3.1(2.71-3.49)		——		——
<60		7.0 (5.56-8.44)	——		——				——		——	
Gender	127	9.2 (7.38-11.02)	0.71(0.51-0.98)	.040	0.84(0.59-1.20)	.337	125	3.7(2.64-4.76)	0.79(0.59-1.07)	.130	0.85(0.61-1.18)	.334
Male	90			——		——	87	3.3(2.70-3.90)		——		——
Female		6.7(4.45-8.95)	——		——				——		——	
Lines of therapy	73	8.9(5.94-11.86)	0.88(0.63-1.23)	.445	1.08(0.74-1.57)	.698	69	4.7(2.68-6.72)	0.75(0.54-1.03)	.072	1.37(0.97-1.92)	-
	144	7.4(5.93-8.87)		——		——	143	3.1(2.66-3.54)		——		.074
<3			——						——		——	——
≥3												
Immune therapy	116	10.3 (7.8-12.8)	0.72(0.51-1.00)	.050	0.66(0.46-0.94)	**.022**	115	3.8(2.86-4.14)	0.85(0.63-1.16)	.309	0.83(0.60-1.16)	.278
	88	7.2 (5.89-8.52)		——		**——**	90	3.5(2.94-4.66)		——		——
Yes			——		——				——		——	
No												
Lung cancer	78	12.3(2.47-22.13)	0.67(0.47-0.95)	.026	0.64(0.41-1.00)	.054	78	3.5(2.22-4.78)	0.80(0.58-1.10)	.173	0.76(0.54-1.14)	.187
Yes	139			——		——	134	3.4(2.79-4.01)		——		——
No		7.2(6.01-8.39)	——		——				——		——	
GI malignancy	83	7.3(6.47-8.14)	1.27(0.91-1.75)	.157	1.16(0.76-1.77)	.484	80	3.5(2.75-4.25)	1.15(0.85-1.56)	.363	1.03(0.71-1.50)	.880
Yes	134	8.6(6.17-11.03)		——		——	132	3.5(2.85-4.15)		——		——
No			——		——				——		——	
Therapy group	82	17.7 (10.2-0.46)	0.30(0.19-0.55)	<.001	0.37 (0.25-25.2)	**<.001**	82	7.0(1.12-12.88)	0.25(0.16-0.38)	<.001	0.30(0.19-0.47)	**<.001**
Matched	47			——		**——**	43	2.3(1.88-2.72)		——		**——**
Unmatched		4.4(3.33-5.47)	——		——				——		——	
Therapy group	82	17.7 (10.2-25.2)	0.42(0.29-0.62)	<.001	0.39 (0.26-0.58)	**<.001**	82	7.0(3.64-14.36)	0.35(0.25-0.51)	<.001	0.35(0.24-0.50)	**<.001**
Matched	88			——		**——**	87	3.0(2.19-3.81)		——		**——**
No marker		6.5(5.07-7.93)	——		——				——		——	

Abbreviations: CI, confident interval; GI, gastrointestinal; OS, overall survival; PFS, progression-free survival; UN, unknown.

PFS was available for analysis in 212 patients (82 in the matched group, 43 in the unmatched group, and 87 in the no-marker group). The median PFS was 7.0 months (95% CI: 1.12-12.88) in the matched group, 2.3 months (95% CI: 1.88-2.72) in the unmatched group (HR, 0.25; 95% CI: 0.16-0.38; *P* < .001), and 3.0 months (95% CI: 1.15-2.85) in the no-marker group (HR, 0.35; 95% CI: 0.25-0.51; *P* < .001; **Figure 3B**).

Multivariate Cox regression showed that among the patients whose cases were deliberated by the MTB, receiving matched therapy was independently associated with favorable PFS relative to the patients who received the unmatched therapy (adjusted HR, 0.37; 95% CI: 0.25-0.55; *P* < .001). Receiving matched therapy was also independently associated with favorable PFS relative to the patients who were not referred to the MTB (adjusted HR, 0.30; 95% CI: 0.19-0.47; *P* < .001).

Among the 185 patients for whom we could determine PFS1 and PFS2, 32.8% of the patients in the matched group achieved a PFS2/PFS1 ratio ≥1.3, compared with 18.4% in the unmatched group and 22.9% in the no-marker group (*P* = .015; **Supplementary Figure 2**).

### Subgroup analysis based on immunotherapy

Among the 217 patients in our final analysis, 116 (53%) received anti-PD-1/PD-L1 agents, either as monotherapy or as part of a combination regimen. We noticed that the proportion of patients who received immunotherapy was not balanced between the matched and no-marker groups (*P* = .013, **Supplementary Table 1**). In addition, we found that across all 3 groups of patients, the risk of all-cause death during the follow-up was significantly lower among those who received immunotherapy than among those who did not (adjusted HR, 0.66; 95% CI: 0.46-0.94; *P =* .019; **Table 2**).

**Figure 4.**
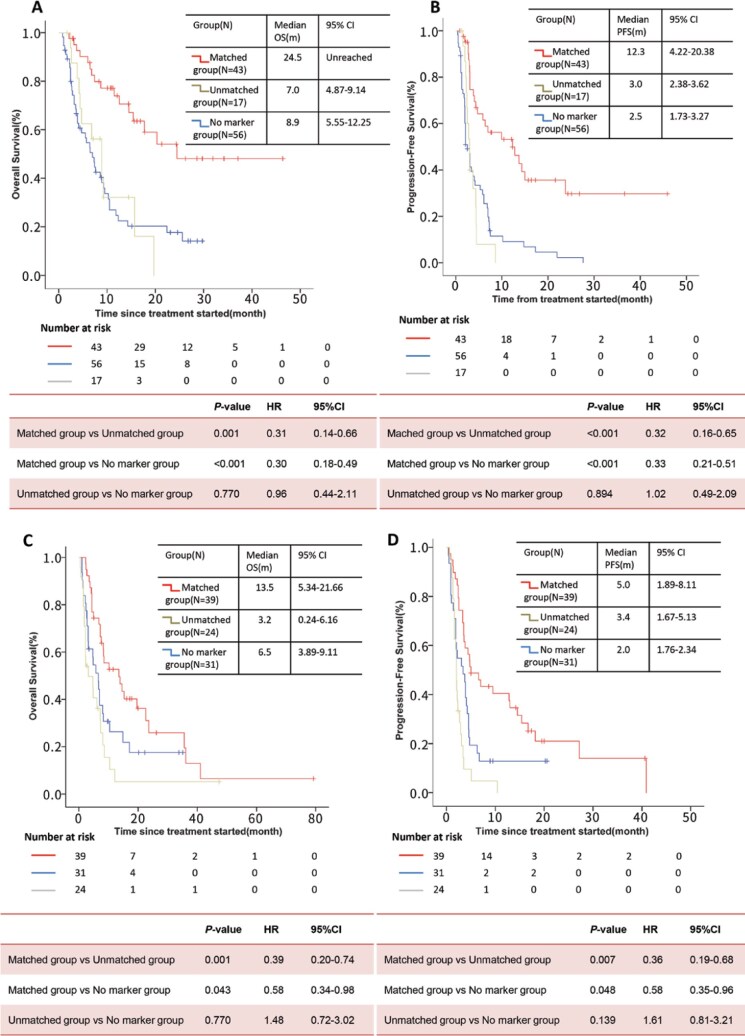
Overall survival and progression-free survival in subanalysis cohortKaplan–Meier curves of (A) overall survival or (B) progression-free survival among the 116 patients who received immunotherapy and the 88 who did not (C–D). “Matching Score,”^30^^,^^31^ indicating that the more targeted targetable mutations, the better overall response observed. However, the EXOMA and the SHIVA studies failed to observe similar results, arguably because few patients in the EXOMA study received combination therapies of targeting agents that may improve the benefit of treatment, and the SHIVA trial restricted itself to include alterations in weak match molecular target agents and hormone therapy.^32^ In the Know Your Tumor program, despite the small percentage of patients who received the MTB-recommended therapy (2%), the use of molecular-targeted therapy significantly improved the prognosis in patients with pancreatic cancer.^33^ In contrast to previous studies, the patients in our study were more heavily treated and in the refractory status, and thus had limited treatment options since tier 1 gene alterations had mostly been exploited. Instead of matching strictly to gene-targeting agents,^13^ MTB-recommended treatments in our experience considered many other relevant issues, including physical condition, organ function, differences of targeting agents, oncogenic driving capacity, tumor heterogeneity, and immune environment.

Therefore, we repeated our comparisons of OS and PFS in the subgroup of 116 patients who received immuno-therapy. The median OS was significantly longer in the matched group than in the unmatched group (HR, 0.31; 95% CI: 0.14-0.66; *P* = .001; **Figure 4A**). The median PFS was 12.3 months (95% CI: 4.22-20.38) in the matched group, significantly longer than 3.0 months (95% CI: 2.38-3.62) in the unmatched group (HR, 0.32; 95% CI: 0.16-0.56; *P <* .001) and 2.5 months (95% CI: 1.73-3.27) in the no-marker group (HR, 0.33; 95% CI: 0.21-0.51; *P <* .001; **Figure 4B**).

Multivariate regression showed that among the patients who received immunotherapy only, receiving the recommended therapy was independently associated with longer OS and PFS relative to the other 2 groups of patients (**Table 3**). In other words, the relationships in OS and PFS among the 3 patient groups were similar regardless of whether we analyzed all patients, the subset of 116 patients who received immunotherapy (**Table 3**), or the subset of 94 patients who did not (Figure 4C–D, **Table 4**).

**Table 3.** Factors associated with OS and PFS in patients who received immunotherapy regimen between different groups.

**Patient characteristics**	**OS**						**PFS**					
	***N***	**Median(month) (95%CI)**	**Univariate Cox model HR (95%CI)**	***P*- value**	**Multivariate Cox model HR (95%CI)**	***P*- value**	***N***	**Median(month) (95%CI)**	**Univariate Cox model HR (95%CI)**	***P*- value**	**Multivariate Cox model HR (95%CI)**	***P*- value**
Age(years)	69	11.5(7.00-16.00)	0.81(0.51-1.31)	.394	0.60(0.36-1.02)	.058	69	4.5(1.67-7.33)	0.99(0.65-1.51)	.964	0.85(0.54-1.35)	.496
≥60	47			——		——	47	3.1(2.55-3.65)		——		——
<60		8.6(6.52-10.69)	——		——				——		——	
Gender	71	12.3(7.63-16.97)	0.65(0.41-1.06)	.082	0.97(0.57-1.65)	.411	71	4.7(2.25-7.15)	0.76(0.50-1.17)	.213	1.03(0.63-1.68)	.902
Male	45			——		——	45	3.1(2.76-3.44)		——		——
Female		8.6 (4.55-9.05)	——		——				——		——	
Lines of therapy	40	8.6(6.52-10.69)	1.1(0.68-1.79)	.700	1.33(0.77-2.30)	.303	40	5.8(2.50-9.10)	1.43(0.92-2.21)	.110	1.77(1.10-2.86)	**.018**
	76	11.5(6.24-15.76)		——		——	76	3.1(2.34-3.86)		——		——
<3			——		——				——		——	
≥3												
Therapy group	43	NR	0.31(0.15-0.67)	.003	0.32 (0.15-0.71)	**.005**	43	12.3(4.22-20.38)	0.82(0.51-1.34)	.436	0.23(0.14-0.40)	**<.001**
	17	8.9(5.55-12.25)		——		**——**	17			——		**——**
Matched			——		——			3.0(2.38-3.62)	——		——	
Unmatched												
Therapy group	43	NR	0.29(0.16-0.52)	<.001	0.25 (0.13-0.46)	**<.001**	43	12.3(4.22-20.38)	0.27(0.13-0.52)	<.001	0.26(0.13-0.53)	**<.001**
	56	7.0(4.87-9.14)		——		**——**	56			——		**——**
Matched			——		——			2.5(1.73-3.27)	——		——	
No marker												
TMB(Mut/Mb)	87	8.9(5.05-12.75)	0.67(0.37-1.23)	1.199	1.18(0.61-2.21)	.621	87	3.7(2.69-4.71)	0.26(0.16-0.44)	<.001	0.91(0.53-1.55)	.725
	29	19.7(3.32-36.08)		——		——	29	4.4(2.41-6.39)		——		——
<10/unknown			——		——				——		——	
≥10												
Lung cancer	36	17.7(6.93-28.47)	0.70(0.41-1.20)	.193	0.86(0.42-1.73)	.663	36	4.5(2.90-4.50)	0.82(0.52-1.30)	.400	0.80(0.44-1.46)	.469
Yes	80			——		——	80	3.7(1.88-4.12)		——		——
No		8.9(6.38-11.42)	——		——				——		——	
GI malignancy	51	9.5(6.91-12.10)	1.11(0.69-1.77)	.672	1.20(0.65-2.22)	.552	51	4.0(3.26-4.74)	1.01(0.67-1.53)	.967	1.40(0.84-2.34)	.932
	65	12.3(6.24-18.36)		——		——	65	3.4(1.88-4.92)		——		——
Yes			——		——				——		——	
No												

Abbreviations: CI, confident interval; GI, gastrointestinal; OS, overall survival; PFS, progression-free survival; TMB, tumor mutaion burden; UN, unknown.

**Table 4.** Factors associated with OS and PFS in patients who received without ICIs regimen between different groups.

**Patient Characteristics**	**OS**						**PFS**					
	***N***	**Median(month) (95%CI)**	**Univariate Cox model HR (95%CI)**	***P*- value**	**Multivariate Cox model HR (95%CI)**	***P*- value**	***N***	**Median(month) (95%CI)**	**Univariate Cox model HR (95%CI)**	***P*- value**	**Multivariate Cox model HR (95%CI)**	***P*** **-value**
Age(years)	41	8.3(6.85-9.75)	0.78(0.48-1.26)	.306	1.10(0.66-1.84)	.715	41	3.7(2.57-4.83)	0.74(0.47-1.15)	.178	1.07(0.65-1.75)	.801
≥60	53	6.8(4.36-9.24)		——		**——**	53	3.1(1.95-4.25)		**——**		**——**
<60			——		**——**				——		**——**	
Gender	51	7.3(3.91-9.69)	0.77(0.49-1.23)	.277	0.70(0.42-1.16)	.169	51	3.5(2.26-4.74)	0.74(0.48-1.16)	.188	0.72(0.45-1.15)	.171
Male	43	6.8(5.92-8.68)		——		**——**	43	3.4(2.76-4.04)		——		**——**
Female			——		**——**				——		**——**	
Lines of therapy	28	8.1(3.23-12.97)	0.77(0.46-1.29)	.321	1.02(0.59-1.75)	.943	28	4(3.05-4.96)	1.47(0.90-2.41)	.127	1.23(0.74-2.04)	.430
	66	6.5(3.92-9.08)		——		**——**	66	2(1.11-2.89)		**——**		**——**
<3			——		**——**				——		**——**	
≥3												
Therapy group	39	13.5(5.34-21.66)	0.38(0.21-0.67)	.001	0.24(0.14-0.40)	**<.001**	39	4.7(3.20-6.20)	0.52(0.29-0.93)	.027	0.46(0.26-0.81)	**.007**
Matched	24			——		**——**	24	2.9(2.01-2.79)		——		**——**
Unmatched		3.2(0.24-6.16)	——		——				——		**——**	
Therapy group	39	13.5(5.34-21.66)	0.57(0.33-0.98)	.044	0.50(0.28-0.90)	**.021**	39	5.0(1.89-8.11)	0.50(0.29-0.85)	.011	0.25(0.14-0.47)	**<.001**
Matched	31			——		**——**	31	3.4(1.67-5.13)		——		**——**
No marker		6.5(3.89-9.11)	——		——				——		**——**	
Lung cancer	40	6.5(4.29-8.71)	0.54(0.33-0.88)	.014	0.52(0.27-0.99)	.107	40	3.5(2.26-4.74)	0.66(0.42-1.05)	.077	0.78(0.43-1.41)	.407
Yes	54	8.5(7.64-9.36)		——		**——**	54	2.9(1.80-4.00)		——		**——**
No			——		**——**				——		**——**	
GI malignancy	30	6.1(3.42-8.78)	1.76(1.09-2.82)	.020	1.44(0.80-2.58)	.220	30	2.5(1.16-3.84)	1.62(1.02-2.56)	.040	1.51(0.85-2.67)	.160
Yes	64	8.1(6.52-9.77)		——		**——**	64	3.5(2.73-4.27)		**——**		**——**
No			——		**——**				——		**——**	

Abbreviations: CI, confident interval; GI, gastrointestinal; OS, overall survival; PFS, progression-free survival; UN, unknown.

## Discussion

To the best of our knowledge, the present study is the largest investigation that assessed survival outcomes in patients receiving MTB-recommended treatments for refractory solid tumors thus far. The study cohort covered a wide range of cancers and patient conditions. More importantly, 66% of the patients received more than 3 previous lines of therapies, reflecting the real-world situation of refractory solid tumor patients. The results showed that MTB-recommended treatment was associated with longer OS and PFS, regardless of whether the treatments included immunotherapy.

We found similar OS between the unmatched group and the no-marker group, suggesting that the presence of actionable genetic alterations per se does not significantly affect survival in patients with refractory solid tumors. In other words, the improved survival in our study can be attributed primarily to whether patients received an MTB-recommended treatment or not. Such a conclusion was supported by multivariate Cox regression that adjusted for several factors known to influence survival. It is important to note that the current study did not include patients with only tier 1 somatic variants of canonical tumor types in the MTB discussion, because standard therapies for treating refractory cases of such cancers have been well-defined. The findings from the current study demonstrated that actionable tier 2 clinical-grade results, including NGS, FISH, and IHC of immune and chemotherapy biomarkers, are considerably meaningful genomic alterations that warrant further deliberation by the MTB. The benefits of MTB-recommended therapies based on tier 2 alterations could be attributed to several factors. First, tier 2 gene alterations across various cancers may support the feasibility of molecular targeted therapy under the basket theory. Second, tier 2 gene alterations may exist as concomitant mutations that potentially affect target therapeutic effectiveness. Third, tier 2 gene alterations may also provide molecular guidance on treatment sensitivity and prognosis prediction to guide alternative treatment decisions, including chemotherapy and immunotherapy. Moreover, tier 2 gene alterations may provide a promising novel target, which is without high-level clinical evidence at present.^26^  *TP53* mutation is the most frequent somatic molecular alteration in cancer patients,^23^^,^^27^ but no therapeutic agents that target *TP53* mutation directly have been approved for use in patients with solid tumors. In the current study, arsenic trioxide, either alone or in combination with chemotherapy, was recommended by the MTB for patients with structural p53 mutants^23^ based on its established efficacy for acute promyelocytic leukemia.^28^^,^^29^ The outcomes could not be assessed due to the small number of patients, and we are currently planning a further study to verify this possibility.

The improved survival of MTB-recommended treatments in the current study is consistent with previous findings. The I-PREDICT and WINTHER trials reported improved efficacy of molecular-guided personalized therapy by the therapy

The improved survival of MTB-recommended therapies in our study did not depend on whether the therapies involved immunotherapy. As a matter of fact, the proportion of patients receiving immunotherapy was much higher among those who were not referred to the MTB, supporting the usefulness of immunotherapy as a salvage treatment. While several actionable events that our MTB considered have been linked to the efficacy of immunotherapy, including PD-L1 expression, TMB, and deficient DNA mismatch repair or high microsatellite instability, and other negative predictors,^34-37^ evidence suggests that a combinational approach can improve survival, regardless of PD-L1 expression and TMB.^38^^,^^39^ Thus, most of our patients receiving immunotherapy also received concomitant chemotherapy, radiotherapy, or antiangiogenic agents. Our study suggests that the use of immunotherapy agents is also best guided by a comprehensive, multifactorial analysis of patient and tumor characteristics by MTB. Future work should continue to search for mechanisms and bio-markers that can clarify—and allow prediction of—response to immunotherapy.^40^

The findings from the current study should be interpreted with caution. First, our study was retrospective and based on a relatively small sample from a single center. Second, our study was designed to investigate refractory solid tumors, and a fundamental limitation of survival analyses was the high rate of missing longitudinal data during follow-up. Third, the study cohort was very heterogeneous, which in turn increased the risk of confounding. Last but not least, some of the patients in the current study ultimately opted for hospice care rather than receiving the MTB-recommended therapy, which may have created a bias in favor of the MTB-recommended therapy.

## Conclusion

The present study supported the feasibility and utility of MTB in managing patients with refractory solid tumors. Patients who adhered to MTB-recommended therapies had longer OS and PFS. This study also highlighted the complex, heterogeneous genetic and molecular alterations among patients with refractory solid tumors, which collectively formed the basis of the benefits of MTB. Continued efforts in this area are worthwhile and will enable more patients with refractory solid tumors to benefit from a precision medicine approach through MTB.

## Supplementary Material

oyaf196_Supplementary_Data

## Data Availability

The datasets generated and analyzed during the current study are available from the corresponding author on reasonable request.
