# Supplementary material for: Impact of molecular tumor board on clinical outcomes in patients with refractory solid tumors: a real-world study
Source: Oncologist. 2025 Jul 1;31(5):oyaf196. doi: 10.1093/oncolo/oyaf196 (PMC13142151; doi:10.1093/oncolo/oyaf196)
Supplement: oyaf196_Supplementary_Data [file oyaf196_supplementary_data.zip › oyaf196_suppl_Supplementary_Figures_1-2.docx]

**Supplementary Figure 1** Distribution of the 30 most frequent genomic alterations in blood NGS.


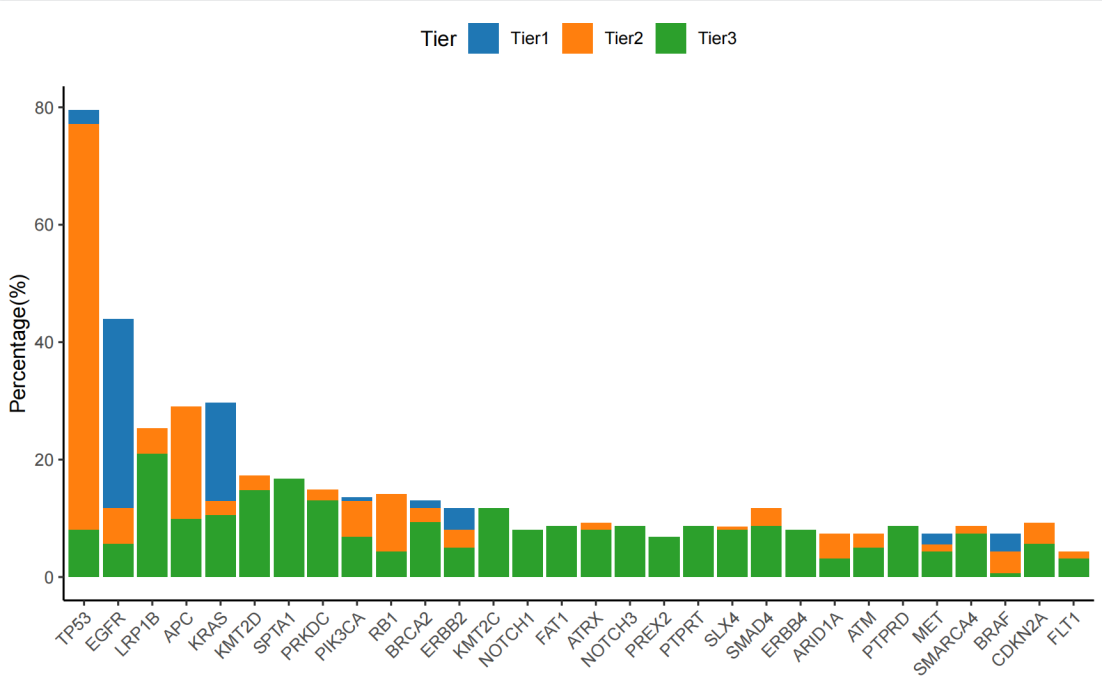


**Supplementary Figure 2** Bar graph of the percentage of PFS2/PFS1 ratio ≥1.3 or ＜1.3 among the three different group.

**Supplementary Table1** Pairwise comparisons of the three treatment groups after patient stratification by the indicated characteristics.

| Patient Characteristics | Chi-square Test *P*-Value  Matched *vs* unmatched therapy | Chi-square Test *P*-Value  Matched *vs* no marker group |
| --- | --- | --- |
| Female *vs* Male | 0.668 | 0.24 |
| Age≤60 *vs* Age>60 | 0.96 | 0.124 |
| ≥3 *vs* <3 lines of previous therapy | 0.474 | 0.416 |
| Recived immunotherapy *or* not | 0.439 | 0.013* |
| Combination therapy *or* monotherapy | 0.788 | 0.115 |
| Lung cancer *or* not | 0.517 | 0.534 |
| Gastrointestinal malignancy *or* not | 0.816 | 0.324 |
